# LATE-LIFE SINGLEHOOD AND WELL-BEING: THE ROLE OF MARITAL STATUS AND SOCIAL RESOURCES

**DOI:** 10.1093/geroni/igae098.2222

**Published:** 2024-12-31

**Authors:** Urmimala Ghose, Rinseo Park, Jacqui Smith, Nilam Ram, Denis Gerstorf

**Affiliations:** Humboldt University Berlin, Berlin, Berlin, Germany; Stanford University, Stanford, California, United States; University of Michigan, Ann Arbor, Michigan, United States; Stanford University, Stanford, California, United States; Humboldt University Berlin, Berlin, Berlin, Germany

## Abstract

Singlehood has long been identified as a potential risk factor for undermining well-being across adulthood, particularly in older ages. However, it is an open question what role other social resources may play. In the current study, we examine whether and how older adults experiencing marital transitions through divorce and spousal bereavement differ from their never married and continuously married peers in their well-being trajectories and in the roles that social support and strain play. We used six waves (2008 – 2018) of longitudinal data from 7,670 participants (Mage = 66 years, 56% women, 75% White) of the Health and Retirement Study (HRS). We also employed synthetic cohort matching to equate the groups on relevant background factors. First results of multi-level models revealed that bereavement trajectories were characterized by a steeper transition-related rise in depressive symptoms than the divorce trajectories, but also by a sharper drop in subsequent years. Both transition groups showed declines in depressive symptoms in the years after the event, yet those who had experienced divorce reached average levels of their continuously married peers after about three years, whereas the bereaved group continued to lag behind even after six years post-spousal loss. Overall, we found little evidence that the relevance of social resources for well-being trajectories differs across marital status groups. As one exception, we found that the predictive effects of strain from family and friends for elevated depressive symptoms was particularly pronounced among those who had experienced spousal loss.

